# Puerperal Infection from the Gonococcus

**Published:** 1907-11

**Authors:** Ellice McDonald

**Affiliations:** Instructor in Obstetrics, New York Post-Graduate Medical School and Hospital; Instructor in Obstetrics, College of Physicians and Surgeons; Clinical Assistant in Obstetrics, Vanderbilt Clinic


### Puerperal Infection from the Gonococcus.

By ELLICE McDONALD, M. D.

Instructor in Obstetrics, New York Post-Graduate Medical School and Hospital;
Instructor in Obstetrics, College of Physicians and Surgeons;
Clinical Assistant in Obstetrics, Vanderbilt Clinic.

(*The Post Graduate*, October, 1907)

INVESTIGATION of the causes of puerperal peritonitis has proved that it is caused by many organisms of which the streptococcus is most prominent, both in severity and frequency. No inconsiderable proportion of cases of puerperal infection are, however, the result of infection with the more uncommon organisms, such as staphylococcus, gonococcus and pneumococcus. The streptococcus, however, is the infecting organism in the majority of cases and was present in 40 per cent. of 498 cases collected by the author,[1] in which the contents of the uterus was studied bacteriologically.

In this study it was interesting to observe that in the earlier cases the gonococcus was but infrequently mentioned, but coincident with improvement in bacteriological technic, the frequency of its isolation from the uterine lochia was much increased. Heretofore widely divergent views have been held in regard to the pathogenicity and frequency of the gonococcus as a cause of puerperal infection. Bumm[2] and Wertheim[3] are inclined to believe that it is not a frequent infecting organism, and Saenger[4] thinks that it may be a more common cause of puerperal and post-puerperal disease.

Most investigations, however, have been wanting in bacteriological thoroughness, and the organism has not been found. The difficulty of cultivation of the organism has been the greatest hindrance to its isolation. Another frequent source of error is that the cultures have most commonly been made soon after the first exhibition of temperature and while the lochia contained much blood and few pus cells. The gonococcus cannot readily be found in smears under these conditions, but is best discovered when numerous pus cells exist and after the infection has advanced somewhat. It was also found, in a study of 17 cases of gonococcus puerperal infection by Stone and the author[5] that the gonococcus was usually readily found in smears from the uterine lochia after the discharge has become purulent.

Of these 17 cases, 12 had rises of temperature to about 100° F., and when the cases of mixed infection were eliminated nine of 14 cases, in which the gonococcus was found in the lochia, had such rises of temperature.* To consider these nine cases first, the fever was mild in three, moderately severe in four, and in two was severe; in five the onset of fever was distributed over the first week; and in one it was as late as the 13th day. The average duration of the fever was 4.1 days, varying from three cases, in which the fever lasted one day, to two cases in which it lasted nine days.

The fever was in all cases very irregular and followed no definite curve. In one case, the infection was very severe, but in the majority of cases this was not so, and temperature curves corresponding to the so-called "sapremic" or septic absorption temperature were more frequently found, the temperature suddenly rising and returning to normal in three or four days.

The three cases of mixed infection had widely varied courses. One of gonococcus and colon infection had fever on the sixth day, which lasted six days and reached 102° three times. Two cases of streptococcus and gonococcus infection also varied. One had a mild course and the other a most severe infection, resulting in death.

These cases show that the gonococcus may be a cause of puerperal infection and may cause severe constitutional disturbance. These findings have been corroborated by Mayer[6] in a study of six cases of such infection and by an investigation of Little[7] into the bacteriology of the recently pregnant uterus. Little found the gonococcus in the lochia in 16 cases. There were 10 cases with a temperature above 100° F.; six cases were in pure culture, two in mixed culture and two doubtful. There were three cases with a rise above 100.6° F.

From this it may be seen that gonococcus infection in the puerperium must not be disregarded. The first course of the infection is not usually acute; but the late manifestations are more dangerous and severe. The common cause of gonococcus infection in the puerperium does not, as a rule, show marked constitutional symptoms, but the danger of extension of the disease to the Fallopian tubes is a very grave one. In nine of the 17 cases such extension was believed to have taken place, as indicated by pain, abdominal rigidity and signs of a mild pelvic infection. In one case this extension was definitely proved by operation.

The disease may extend still further and cause general peritonitis, as is shown by a case reported by the author[8] in which a diffuse peritonitis resulted from gonococcus infection in the puerperium. The organism was recovered from the peritoneum at autopsy.

The character of the lochia in gonococcus infection in the early part of the puerperium remains unchanged, but after the fifth day it becomes more and more purulent, until it is replaced by a purulent discharge. This discharge, resultant from the endometritis, usually persists for some weeks. During this time the gonococcus may be readily isolated.

An interesting fact in connection with this infection is the malnutrition and intestinal disturbances to which babies of infected mothers are subject. Of fourteen babies, three died, and the majority of the others lost weight. These facts in regard to the morbidity of the babies have been confirmed by Lobenstine[9] in fifty cases.

The influence of labor upon a preëxistent gonococcus infection is most marked. The softened tissues and large, raw surface of the puerperal uterus offer a splendid culture ground for the organism. It usually extends by mucous membrane, but may penetrate the softened uterine muscle. Extension to the Fallopian tubes is the common result and late disturbances are the rule.

Two cases of the seventeen came to operation for purulent salpingitis and others have not been traced.

The gravity of this infection exists not so much in its prime infection and immediate constitutional disturbances as in the more remote result of tubal and pelvic disease some time after the puerperium. It is a well known fact that streptococcus infection results in slight anatomic alterations of the pelvic organs after recovery from the infection, but the reverse is true of gonococcus infection where marked alteration of tissue is the rule and spontaneous recovery from pelvic disease from this cause is the exception.

---

1. McDonald, *Montreal Med. Journal*, August, 1905.
2. Bumm, Veit's *Handbuch der Gynekologie*, Band 1.
3. Wertheim, *Verhandl. der Deutsch. Gesellsch. f. Gynekologie*, 1895.
4. Saenger, *Ibid.*, 1886, 177.
5. Stone and McDonald, *Surgery, Gynecology and Obst.*, February, 1906.
6. Mayer, *Mon. f. Geb. u. Gyn.*, 1907.
7. Little, *Amer. Jour. of Obst.*, December, 1906.
8. McDonald, *Puerperal General Peritonitis, Annals of Surgery*, February, 1907.
9. Lobenstine, *Bull. of Lying-in Hospital*, 1905.

